# Comparative evaluation of symmetry, dosimetry, and toxicity in prostate cancer EBRT with spacing techniques

**DOI:** 10.3389/fonc.2025.1668726

**Published:** 2025-12-15

**Authors:** Yossi Ben-Dor, Aaron Shachar, Eleonora Kuptzov, Salem Billan, Tomer Charas

**Affiliations:** Radiotherapy Institute, Oncology Division, Rambam Health Care Campus, Haifa, Israel

**Keywords:** EBRT = external beam radiation therapy, prostate spacers, dosimetry, spacer geometry, toxicity, spacer symmetry

## Abstract

**Introduction:**

The proximity of the rectum to the prostate in radiation therapy for prostate cancer presents a significant dosimetric challenge, leading to high rectal doses and resulting in detrimental side effects. Perirectal tissue spacing reduces rectal dose and gastrointestinal toxicities by mechanically separating these organs. We retrospectively compared balloon and PEG hydrogel spacers, focusing on spacer geometry, symmetry, rectal dosimetry, and GI/GU toxicity.

**Methods:**

Sixty-seven men with localized prostate cancer treated with EBRT were analysed (balloon = 33; PEG hydrogel = 34). Symmetry was graded on axial CT at apex, mid-gland, and base with a five-tier midline scale (SYM-1 = optimal). Anteroposterior, laterolateral, and craniocaudal separations were measured. Rectal V60%–V100% were taken from dose–volume histograms. Acute (≤90 d) and late (>90 d) GI/GU toxicities were scored (CTCAE v4.0). Two-sided p ≤ 0.05 was significant.

**Results:**

Optimal symmetry occurred in 33% (balloon) vs 14% (PEG hydrogel); asymmetry SYM-4/5 in 27% vs 24% (p = 0.21). At the apex, balloon spacers consistently created measurable separation, whereas 3 patients (9%) with PEG hydrogel demonstrated complete absence of spacing. Mean anteroposterior separation was larger with balloon at all levels (p < 0.001). Laterolateral differed inferiorly (2.4 cm vs 1.9 cm; p = 0.01). Craniocaudal length averaged 4.8 cm vs 4.3 cm (p < 0.001). Rectal V60–V100% showed no significant differences. Acute toxicity was low: GI grade 1 in 6% (balloon) vs 0%, with one grade 3 GI in PEG hydrogel; GU grade 1 in 13% vs 29%, grade 2 in 10% vs 7%. Late events: GI grade 2 in 0% vs 7%; GU grade 3 in one patient per cohort (~3%); other late toxicities mild and similar.

**Conclusion:**

The balloon spacer achieved greater, more uniform separation including improved apical symmetry, and showed fewer early GI events and lower mild acute GU rates, while rectal doses remained comparable. Prospective studies with longer follow-up are needed to confirm long-term benefit.

## Introduction

Perirectal spacers are used in external beam radiation therapy (EBRT) for prostate cancer to reduce radiation-induced rectal toxicity by increasing the separation between the prostate and rectum, a primary organ at risk (OAR) (1). Even with advances in treatment planning and delivery, the proximity of the rectum to the prostate causes unavoidable radiation exposure, increasing the risk of gastrointestinal (GI) toxicity (2). Spacer placement displaces the rectum from high-dose regions, reducing radiation exposure and makes treatment more tolerable (3, 4).

Several spacer technologies are available (3–5). The PEG hydrogel (SpacerVue, Boston Scientific Inc., Bedford, MA) has been widely used and has demonstrated reductions in rectal dose exposure and associated toxicity (2). The placement technique and anatomical limitations associated with the PEG hydrogel spacer can lead to variability in spacer distribution, as it is typically injected at the midgland and directed cranially, which may result in reduced separation at the apex relative to the midgland and base. The BioProtect Balloon Implant System (BioProtect Ltd., Tzur Yigal, Israel) achieves separation through controlled inflation, potentially offering more uniform anatomical displacement (4, 6–8). Rectal spacer symmetry has been identified as a critical factor in optimizing rectal dose reduction and a published symmetry model that quantifies spacer placement quality and its relationship with rectal dose reduction, demonstrated that asymmetrical spacer positioning reduces its protective effect. Asymmetrical placement has been associated with higher rectal radiation exposure (9).

This work is aimed at comparing between two spacer technologies, the PEG hydrogel spacer and the BioProtect balloon, which are routinely used in our institution. Our work included analysis of symmetry and geometry, rectal dosimetry, as well as GI and genitourinary (GU) toxicity outcomes, using the published symmetry methodology (9).

## Materials and methods

After IRB approval, a retrospective case-matched analysis included patients with prostate cancer treated with EBRT using either a balloon or PEG hydrogel spacer. For balloon cases, saline mixed with iodinated contrast material was used to enhance the balloon’s visualization on the planning CT and cone-beam CT scans (7, 10). CT scans were done with high-resolution CT scans at ≤ 2 mm slice thickness. Data were collected from institutional records including demographics, dosimetry, spacer volume and geometry, symmetrical alignment according to CT images, and toxicity outcomes.

Two separate methods for spacing utility were used. Spacer symmetry was assessed using the model described by Fischer et al. (9) evaluating spacer positioning at midsagittal plane across three axial levels (midgland, 1 cm superior, and 1 cm inferior). Each slice was scored based on midline placement or lateral deviation, with an overall symmetry score (SYM-1 to SYM-5) assigned. Spacer geometry was assessed using a slightly modified version of Grossman et al. (11), by measuring height, width, and length at a single midline reference point. Height (anteroposterior dimension) and width (laterolateral dimension) were measured at the apex, midgland, and base, while length (craniocaudal dimension) was measured continuously from the most superior point of the spacer to the most inferior one.

Dosimetry plans were evaluated for rectal dose exposure. The clinical target volume (CTV) included the entire prostate with or without the inclusion of the seminal vesicles, at the discretion of the treating physician. Planning target volumes (PTV) were a 5- or 7-mm expansion of the entire CTV, regardless of spacer type placed. The prescription dose was planned to allow delivery to ≥95% of the PTV and 100% of the CTV. Case matching was based on prescribed dose.

Acute and late GI and GU toxicities were rated according to the Common Terminology Criteria for Adverse Events (CTCAE) v4.0. This included frequency/urgency, hesitancy, incontinence, nocturia, dysuria, and hematuria for GU, and urgency, rectal pain/pressure, bleeding, mucus discharge, tenesmus, and diarrhea for GI. Acute toxicity included events that happened within 90 days after radiation treatments, while late toxicity occurred after 90 days. Patients were evaluated weekly during treatment and at 3- and 6-month follow-ups.

Descriptive statistics was used to characterize the variables of interest. Case matching was done by paring of patients who received the same radiotherapy dose as follows: SBRT, moderately hypofractionated EBRT, or conventionally fractionated EBRT, and target set (pelvic field presence and any boost). Cases with unmatched dose prescriptions were excluded to minimize potential confounding related to dosimetry variability.

Each of the variables was checked for normality of distribution using Shapiro-Wilk test. Patient and baseline characteristics were compared with the Fisher exact test and the Wilcoxon rank sum test, where appropriate. Statistical significance between SYM1 and all other symmetry groups was evaluated with student *t* test. Continuous variables were summarized with means and standard deviations and shown to approximate a normal distribution with a Shapiro-Wilk normality test. A p-value of ≤ 0.05 was considered to be statistically significant. All analyses were performed using the SPSS Statistics package (v. 24.0).

## Results

A total of 67 patients were included in the study, with 33 in the balloon group and 34 in the PEG hydrogel group. Patient, tumor, and volume characteristics are summarized in **Table 1**. The Balloon and PEG hydrogel groups were comparable in age, PSA levels, T-stage distribution, and Gleason score, with no significant differences observed. Spacer volume was larger in the balloon group (14.0 ± 3.6 cc) compared to PEG hydrogel (9.9 ± 4.3 cc, p < 0.001). Prostate, rectal, bladder and PTV volumes showed no significant differences between groups.

**Table 1:** Patient Demographic and Baseline Characteristics.

Characteristic	Balloon (n=33)	PEG Hydrogel (n=34)	P-Value
Age (Years)			0.72
Mean (SD)	73.3 (5.1)	72.6 (5.7)	
Median (Q1, Q3)	74.0 (71.0, 76.0)	73.0 (68.2, 76.0)	
Pretreatment PSA (ng/mL)			0.89
Mean (SD)	13.5 (15.96)	10.9 (14.0)	
Median (Q1, Q3)	8.0 (5.8, 10.0)	7.0 (5.8, 9.9)	
T Stage, n (%)			0.93
T1a	0	1 (2.9)	
T1c	22 (66.7)	20 (58.8)	
T2a	1 (3.0)	3 (8.8)	
T2b	1 (3.0)	1 (2.9)	
T2c	1 (3.0)	1 (2.9)	
T3a	3 (9.1)	3 (8.8)	
T3b	2 (6.1)	4 (11.8)	
T4	2 (6.1)	1 (2.9)	
M1b	1 (3.0)	0 (0)	
Gleason Score			0.97
3+3	1 (3.0%)	1 (2.9%)	
3+4	6 (18.2%)	9 (26.5%)	
4+3	12 (36.4%)	10 (29.4%)	
4+4	9 (27.3%)	8 (23.5%)	
4+5	5 (18.2%)	5 (14.7%)	
5+3	0 (0.0%)	1 (2.9%)	
Prostate Volume (cc)			0.41
Mean (SD)	45.0 (18.1)	49.1 (20.0)	
Median	42.1	43.2	
Min, Max	17.59, 83.3	20.6, 108.6	
Rectal Volume (cc)			0.26
Mean (SD)	84.4 (47.9)	73.8 (26.3)	
Median	70.3	64.3	
Min, Max	35.3, 216.2	32.7, 145.2	
Bladder Volume (cc)			0.15
Mean (SD)	157.0 (72.7)	132.0 (67.7)	
Median	151.5	119.4	
Min, Max	57.9, 342.0	40.3, 281.2	
PTV (Inc. Seminal Vesicles) Volume (cc)			0.26
Mean (SD)	125.7 (40.4)	134.6 (38.9)	
Median	114.8	128.7	
Min, Max	62.2, 225.0	65.3, 215.9	
Spacer`s Volume (cc)			<0.001
Mean (SD)	14.0 (2.9)	9.9 (3.2)	
Median	13.7	10	
Min, Max	6.9, 19.5	2.3, 17.5	

### Spacers utility

Spacer symmetry was scored according to **Table 2** as reported by Fischer-Valuck (9) and the comparative results between the studies groups are fully detailed in **Table 3**.

**Table 2:** Symmetry scoring metrix.

(A) Axial slice spacers symmetry scoring method	
Score	Classification
1	Spacer positioned at the prostate midline
2L/ 2R	Spacer positioned asymmetry 1 cm from the midline, to the left or right
3L/ 3R	Spacer positioned asymmetry ≥2 cm from the midline, to the left or right
0	No spacer in slice
(B) Overall symmetry score method	
SYM-1	All 3 slices are symmetrical.
SYM-2	1 slice shows a 1 cm lateral distribution. 2 out of 3 slices are symmetrical
SYM-3	2 slices show a 1 cm lateral distribution. 1 out of 3 slices is symmetrical
SYM-4	All 3 slices show 1 cm lateral distribution
	2 slices show a 1 cm lateral distribution and 1 slice show 2 cm lateral distribution
	2 slices show a 1 cm lateral distribution and 1 slice with no spacer
SYM-5	All 3 slices show 2 cm lateral distribution
	2 slices show a 2 cm lateral distribution and 1 slice show a 1 cm lateral distribution
	2 slices show a 2 cm lateral distribution and 1 slice with no spacer

**Table 3:** Symmetry comparison.

Symmetry Scores Per Slice				
Symmetry Score	Axial slice	Balloon (n=33)	PEG Hydrogel (n=34)	p-value
Inferior				0.04
	1 (n, %)	18 (54.5)	15 (44.1)	
	2L (n, %), 2R (n, %)	8 (24.2), 3 (9.1)	7 (20.6), 8 (23.5)	
	3L (n, %), 3R (n, %)	0 (0.0), 4 (12.1)	1 (2.9), 0 (0.0)	
	0 (n, %)	0 (0.0)	3 (8.8)	
Midgland				0.37
	1 (n, %)	18 (54.5)	18 (52.9)	
	2L (n, %), 2R (n, %)	6 (18.2), 6 (18.2)	9 (26.5), 7 (20.6)	
	3L (n, %), 3R (n, %)	0 (0), 3 (9.1)	0 (0.0), 0 (0.0)	
	0 (n, %)	0 (0.0)	0	
Superior				0.47
	1 (n, %)	10 (30.3)	12 (35.3)	
	2L (n, %), 2R (n, %)	5 (15.2), 9 (27.3)	9 (26.5), 5 (14.7)	
	3L (n, %), 3R (n, %)	1 (3.0), 4 (12.1)	1 (2.9), 1 (2.9)	
	0 (n, %)	4 (12.1)	6 (17.6)	
Overall Patient-level Symmetry scoring				
0.21				
Optimal Symmetry (SYM-1)		11 (33.3)	5 (14.7)	
Moderate Symmetry (SYM-2-3)		13 (39.4)	21 (61.8)	
Asymmetry (SYM-4-5)		9 (27.3)	8 (23.5)	

Among the 67 patients evaluated, centered spacer placement at the apical slice (score 1) was observed in 18 of 33 balloon cases (54.5%) compared to 15 of 34 PEG hydrogel cases (44.1%), reaching statistical significance (p = 0.042). Notably, none of the balloon cases showed absent spacers at the apex (score 0), whereas this was observed in 8.8% of PEG hydrogel cases. At the mid-gland level, score 1 symmetry was nearly identical between groups—18 of 33 balloon cases (54.5%) versus 18 of 34 PEG hydrogel cases (52.9%)—with no score 0 occurrences in either group (p = 0.366). At the base, score 1 was observed in 10 of 33 balloon cases (30.3%) and 12 of 34 PEG hydrogel cases (35.3%) (p = 0.470), while score 0 was present in 12.1% and 17.6% of cases, respectively. On a per-patient basis, perfect symmetry across all slices (SYM-1) was achieved in 11 of 33 (33.3%) balloon patients and 5 of 34 (14.7%) PEG hydrogel patients; moderate symmetry (SYM-2–3) occurred in 13 (39.4%) versus 21 (61.8%), and asymmetry (SYM-4–5) in 9 (27.3%) versus 8 (23.5%), p= 0.210.

Spacer geometry results are detailed in **Table 4**. Balloon spacers produced greater antero-posterior separation than the PEG hydrogel spacer at each level of the prostate (inferior, mid-gland, superior, all p<0.001). While latero-lateral symmetry was similar at the mid-gland and superior slices for both spacers, the inferior slice showed better width with the balloon spacer (p = 0.019). The cranio-caudal extension of the spacer was also larger with the balloon spacer (p<0.001), **Table 4**.

**Table 4:** Spacer Geometry.

Characteristic	Balloon (N = 33)	PEG Hydrogel (N = 34)	p-value
Antero-posterior (cm)			
Inferior			< 0.001
Mean (SD)	1.7 (0.4)	1.1 (0.5)	
Median (Q1, Q3)	1.8 (1.5, 1.9)	1.2 (0.8, 1.4)	
Mid-gland			< 0.001
Mean (SD)	1.7 (0.4)	1.2 (0.3)	
Median (Q1, Q3)	1.7 (1.5, 1.9)	1.2 (1.1, 1.4)	
Superior			< 0.001
Mean (SD)	1.4 (0.6)	0.6 (0.6)	
Median (Q1, Q3)	1.5 (1.2, 1.9)	0.6 (0.0, 1.1)	
Latero-lateral (cm)			
Inferior			0.02
Mean (SD)	2.4 (0.6)	1.9 (0.9)	
Median (Q1, Q3)	2.5 (2.1, 2.7)	2.0 (1.5, 2.5)	
Mid-gland			0.92
Mean (SD)	2.6 (0.5)	2.6 (0.7)	
Median (Q1, Q3)	2.6 (2.4, 3.0)	2.7 (2.0, 3.1)	
Superior			0.72
Mean (SD)	2.1 (1.0)	1.9 (1.3)	
Median (Q1, Q3)	2.4 (1.7, 2.8)	2.0 (1.0, 2.8)	
Cranio-Caudal (cm)			
Midsagittal			<0.001
Mean (SD)	4.8 (0.4)	4.3 (0.7)	
Median (Q1, Q3)	4.9 (4.7, 5.0)	4.3 (4.0, 4.7)	

### Rectal dosimetry

After case matching for similar radiation protocols, 62 patients were included, as detailed in **Table 5**.

**Table 5:** Dose Matching and Unmatched Cases.

Category	Prostate dose (cGy)	Pelvic LN dose (cGy)	Boost dose (cGy)	Additional level (cGy)	n (Balloon)	n (PEG)
Prescriptions-matched						
SBRT	3600				3	3
	3750				2	1
	4000				9	11
	3750	2500			1	1
Moderate hypofractionated EBRT	6000				4	1
	4000	4600			2	2
	6000	4400			3	2
	6000	4400	5400		1	1
	6000	4400	6800		1	1
Conventionally fractionated EBRT	7000				4	5
	7000	5040			1	1
Unmatched Prescriptions						
Moderate hypofractionated EBRT	6300				1	0
	6000	4400	6900	5400	0	1
Conventionally fractionated EBRT	7000	5040	6300		0	2
	7000	5040	7840		0	1

SBRT, stereotactic body radiotherapy; EBRT, external beam radiotherapy; LN, lymph node.

All dosimetry values included in our anaylsis, were not statistically significant between the two groups (**Table 6**). Dosimetric values were chosen as these are the most commonly used in the clinic and were reported in previous studies evaluating rectal spacers.

**Table 6:** Rectal Dosimetry Parameters by spacer type.

Parameter	Balloon (N=32)	PEG Hydrogel (N=30)	Absolute Decline	Relative Decline (%)	p value
V60%					p = 0.66
Mean (SD)	10.8 (9.3)	11.3 (8.5)	-0.5 (-0.8)	-4.4 (-9.4)	
Median (Q1, Q3)	9.7 (3.3–14.8)	8.5 (5.3–15.2)	1.2	12.3	
V70%					p = 0.35
Mean (SD)	6.1 (5.1)	7.2 (6.2)	-1.1 (-1.1)	-15.3 (-17.7)	
Median (Q1, Q3)	4.7 (1.5–9.6)	5.0 (2.6–10.7)	-0.3	-6.4	
V80%					p = 0.34
Mean (SD)	3.3 (3.2)	4.3 (4.3)	-1.0 (-1.1)	-23.3 (25.6)	
Median (Q1, Q3)	2.6 (0.4–5.7)	2.5 (1.1–6.7)	0.1	3.8	
V90%					p = 0.38
Mean (SD)	1.6 (1.8)	2.0 (2.5)	-0.4 (-0.7)	-20.0 (-28.0)	
Median (Q1, Q3)	0.8 (0.0–2.7)	0.8 (0.2–2.9)	0	0	
V100%					p = 0.67
Mean (SD)	0.2 (0.7)	0.1 (0.3)	0.1 (0.4)	35.1 (53.7)	
Median (Q1, Q3)	0.0 (0.0–0.2)	0.0 (0.0–0.2)	0	0	

### Safety and toxicity

There were no procedure related complications. None of the patients experienced adverse reaction or irritations attributed to the use of contrast-media following the balloon decomposition. No differences were found between the two groups regarding acute or late GI toxicity. Of note, acute GU toxicity grade 1 was 13.3% vs 28.6% in the balloon and PEG hydrogel groups, respectively, however this was not statistically significant (p = 0.36), **Table 7**.

**Table 7:** Toxicity by spacer type.

Toxicity / Grade	Balloon (N=32)	PEG Hydrogel (N=30)	p value
Gastrointestinal (GI) Toxicity			
Acute			>0.5
Grade 1	2 (6.7%)	0 (0.0%)	
Grade 2	0 (0.0%)	0 (0.0%)	
Grade 3	0 (0.0%)	1 (3.6%)	
Late			>0.5
Grade 1	1 (3.3%)	1 (3.6%)	
Grade 2	0 (0.0%)	2 (7.1%)	
Grade 3	0 (0.0%)	0 (0.0%)	
Genitourinary (GU) Toxicity			
Acute			0.36
Grade 1	4 (13.3%)	8 (28.6%)	
Grade 2	3 (10.0%)	2 (7.1%)	
Grade 3	0 (0.0%)	1 (3.3%)	
Late			>0.5
Grade 1	7 (23.3%)	8 (28.6%)	
Grade 2	4 (13.3%)	3 (10.7%)	
Grade 3	1 (3.3%)	2 (6.6%)	

## Discussion

Rectal spacers are used in prostate cancer radiotherapy to increase prostate-rectal separation and reduce rectal dose exposure, thereby minimizing treatment-related toxicity (2, 12, 13). While their dosimetric advantages are well established across radiotherapy modalities (2, 4) spacers might be different in their symmetry and geometry, which may play a key role in their effectiveness. Previous PEG-hydrogel studies linked greater asymmetry with less rectal sparing (9), Quantitative assessments highlight the value of midline and mid-gland centering (14), and the Spacer Quality Score associates higher separation quality across base, midgland, and apex with lower rectal dose and reduced late toxicity (11).

These observations are aligned with published randomized evidence. The PEG hydrogel pivotal trial reported a reduction in rectal dose with early safety comparable to controls (3), and its 3-year follow-up showed sustained improvement in bowel-related QOL and lower late rectal toxicity (2). The multicenter randomized balloon trial reported a reduction in mean rectal V70 from 7.0% pre-implant to 1.1% post-implant and showed low rates of early rectal, device, and procedure-related adverse events compared with control, with perirectal separation remaining stable during EBRT (4). Moreover, in this study, the balloon height was found to be similar at base 2.0cm(± 0.40), mid-gland 2.0cm(± 0.44), and apex 1.8cm(± 0.38) locations, respectively, implying symmetrical spacing from prostatic apex to the base. Consistent with these data, in another prospective study (10) the reported median relative reduction in rectal V70 was 91.4%. In our cohort, V70 remained low with both spacer types and did not differ significantly. This study assessed spacer symmetry and geometric distribution, revealing significant differences between balloon and PEG hydrogel spacers in some aspects. The balloon spacer achieved higher symmetry score at the inferior (apex) location, meaning better centralized location at this level, as well a greater apical prostate–rectum separation. This finding is clinically meaningful, as several studies emphasize the specific importance of apical spacing. King et al. (15) showed that greater apical spacing with a PEG hydrogel spacer was associated with improved rectal dosimetry and smaller decline in acute post-treatment bowel symptoms. It should be noted, however, that King et al. (15) defined the apical plane as the most inferior contoured slice of the prostate, whereas our analysis followed the Fischer model (9), in which the inferior slice is defined 1 cm below the midgland with additional evaluation at the anatomical apex.

High-grade asymmetry (SYM-4-5) reflects marked midsagittal lateralization across levels. PEG hydrogel cohort, asymmetry was more frequent inferiorly, reflecting the well-recognized challenge of achieving uniform hydrogel coverage at the apex (16). Limited needle reach and lower injection volume toward the distal tip often leave the apical region partially uncovered or unevenly expanded, resulting in greater lateralization at that level (9, 11, 16). In the balloon cohort, high-grade asymmetry most plausibly reflects premature final deployment before full axial midline confirmation (7). If detachment occurs without a brief deflate–recenter–reinflate to correct subtle off-axis seating, the balloon’s geometry is fixed and minor misalignment persists as SYM-4-5.

Fukumitsu et al. identified a significant association between apical separation and reductions in high-dose rectal exposure (V50–60 Gy) (17). Narukawa et al. also confirmed that increased apical spacing significantly reduces rectal V30–60, highlighting its impact on dose distribution (18). These data support prioritizing apical separation to enhance rectal protection.

It was also shown that the balloon spacer had a larger antero-posterior interspace separation throughout all prostate levels. This difference reflects the balloon’s controlled, predefined structure that ensures consistent segregation along the entire prostate length with deployment, compared to the PEG hydrogel’s susceptibility to uneven distribution during injection (13). The balloon insertion method mitigates anatomical challenges, allowing uniform and sufficient interspace thickness, as quantified by Grossman et al. (11) quality score. Accordingly, uniform antero-posterior separation across prostate levels would be expected to lower high-dose rectal volumes, including V70. In the phase II multicenter study, the mean ± SD antero-posterior distance was 0.22 ± 0.20 cm before insertion and 2.47 ± 0.47 cm after insertion, and this separation was maintained during radiotherapy with concomitant reductions in rectal dose (19). In our study, the cranio-caudal extension of the balloon was significantly longer than that of the PEG hydrogel. This in turn might lead to better separation in larger prostates, although we did not evaluate this as our cohort was too small and prostate volumes were similar. Larger prostate volume has been associated with reduced spacer effectiveness and increased rectal radiation exposure. Narukawa et al. (18) reported that patients with larger prostates were less likely to achieve ≥7.5 mm apical separation, resulting in significantly higher rectal doses. King et al. (15) similarly found prostate volume to be a predictor of increased rectal V30.

This study has several limitations that should be noticed when interpreting the results. The retrospective, single-center design raises the risk of selection bias and reduces the applicability of the findings, particularly due to the relatively small sample size and short follow-up period, which may miss late or rare adverse events. Additionally, using manual contouring, different radiation protocols, and varying dosage combinations, along with the lack of blinding in outcome assessment, could lead to confounding factors and observer bias. Spacer stability was only checked at the time of simulation, so any changes like migration or deformation during treatment were not assessed. Future prospective randomized controlled trials (RCTs) are needed to confirm these findings and provide stronger evidence.

## Conclusion

The balloon spacer showed better overall symmetry, especially at the apex. It also provided more consistent anterio-posterior separation and longer cranio-caudal extension compared to the PEG hydrogel spacer. These spatial benefits may improve rectal protection, particularly in anatomically difficult areas. Despite these differences, no significant variation was found between groups in rectal dose exposure or GI/GU toxicity rates after dose matching. The lack of pre-insertion dosimetric data, differences in radiation delivery, small group size, and limited follow-up likely limited the ability to find clinical differences. Still, the early results support technical feasibility, positional stability, and comparable performance of the balloon spacer. This highlights the need for future studies to see if its geometric benefits lead to long-term clinical advantages.

## Data Availability

The original contributions presented in the study are included in the article/supplementary material. Further inquiries can be directed to the corresponding author.
